# Indigenous Georgian Wine-Associated Yeasts and Grape Cultivars to Edit the Wine Quality in a Precision Oenology Perspective

**DOI:** 10.3389/fmicb.2016.00352

**Published:** 2016-03-22

**Authors:** Ileana Vigentini, David Maghradze, Maurizio Petrozziello, Federica Bonello, Vito Mezzapelle, Federica Valdetara, Osvaldo Failla, Roberto Foschino

**Affiliations:** ^1^Department of Food, Environmental and Nutritional Sciences, Università degli Studi di MilanoMilan, Italy; ^2^Institute of Horticulture, Viticulture and Oenology, Agricultural University of GeorgiaTbilisi, Georgia; ^3^Centro di Ricerca per l’Enologia, Consiglio per la Ricerca in Agricoltura e l’Analisi dell’Economia AgrariaAsti, Italy; ^4^Department of Agricultural and Environmental Sciences, Università degli Studi di MilanoMilan, Italy

**Keywords:** Georgian grapevine cultivar, wine volatile compounds, yeast biodiversity, GC-FID analysis, Goruli Mtsvane, Saperavi, *Torulaspora delbrueckii*, *Kluyveromyces marxianus*

## Abstract

In Georgia, one of the most ancient vine-growing environment, the homemade production of wine is still very popular in every rural family and spontaneous fermentation of must, without addition of chemical preservatives, is the norm. The present work investigated the yeast biodiversity in five Georgian areas (Guria, Imereti, Kakheti, Kartli, Ratcha-Lechkhumi) sampling grapes and wines from 22 different native cultivars, in 26 vineyards and 19 family cellars. One hundred and eighty-two isolates were ascribed to 15 different species by PCR-ITS and RFLP, and partial sequencing of D1/D2 domain 26S rDNA gene. *Metschnikowia pulcherrima* (*F*’ = 0.56, *I*’ = 0.32), *Hanseniaspora guilliermondii* (*F*’ = 0.49, *I*’ = 0.27), and *Cryptococcus flavescens* (*F*’ = 0.31, *I*’ = 0.11) were the dominant yeasts found on grapes, whereas *Saccharomyces cerevisiae* showed the highest prevalence into wine samples. Seventy four isolates with fermentative potential were screened for oenological traits such as ethanol production, resistance to SO_2_, and acetic acid, glycerol and H_2_S production. Three yeast strains (*Kluyveromyces marxianus* UMY207, *S. cerevisiae* UMY255, *Torulaspora delbrueckii* UMY196) were selected and separately inoculated in vinifications experiments at a Georgian cellar. Musts were prepared from healthy grapes of local varieties, Goruli Mtsvane (white berry cultivar) and Saperavi (black berry cultivar). Physical (°Brix) and microbial analyses (plate counts) were performed to monitor the fermentative process. The isolation of indigenous *S. cerevisiae* yeasts beyond the inoculated strains indicated that a co-presence occurred during the vinification tests. Results from quantitative GC-FID analysis of volatile compounds revealed that the highest amount of fermentation flavors, such as 4-ethoxy-4-oxobutanoic acid (monoethyl succinate), 2-methylpropan-1-ol, ethyl 2-hydroxypropanoate, and 2-phenylethanol, were significantly more produced in fermentation conducted in Saperavi variety inoculated with *K. marxianus*, whereas other aromatic compounds like 3-methylbutyl acetate, ethyl hexanoate and dihydrofuran-2(3H)-one (γ- butyrolactone) showed a higher content in Goruli Mtsvane variety samples fermented by *S. cerevisiae*. The selected yeast strains have proved to be promising for enhancing the flavor potential in low aromatic Georgian cultivars. This work intends to be a knowledge contribution for a precision oenology toward the strategic concept of “one grape variety-one yeast”.

## Introduction

The domestication of grapevine (*Vitis vinifera* L.) occurred somewhere in the geographic region including Eastern Anatolia, South Caucasus and Western Asia in the VI millennium B.C. and it was likely consecutive of the development of the wine-making technologies originally based on wild grapes and other juicy fruits (McGovern, 2003; Forni, 2012; Batiuk, 2013). Georgian people, a proud population tied to their traditions, has always cultivated grapes and produced wines in every village and family, as an ancient local proverb says, “a good father makes a good wine”. Indeed, this country is one of the homeland of the wild species *Vitis vinifera* ssp. *silvestris*, the ancestor of the cultivated grapevine *Vitis vinifera* ssp. *sativa.* The presence of numerous native varieties in Georgia evidences a high degree of intraspecific diversity as a consequence of the heterogeneity of the environments that passing by a Mediterranean climate near the Black Sea, to a subtropical one in the South or continental in the mountainous Northern territories (This et al., 2006; Ghlonti, 2010; Chkhartishvili and Maghradze, 2012).

According to the long Georgian tradition in winemaking, which is still practiced in Kakheti area, oenologists make wine in the traditional “*qvevri*”, a big-size clay vessel put underground and inside coated of beeswax and with long time (until 6 month) of maceration. It is worth mentioning that neither commercial cultures nor sulfur dioxide are used in any of the familiar wineries. Sometimes winemakers use fumigation to sanitize the clay vessel. Nowadays, different types of wine are made in *qvevri*: (i) the “Kakhetian style”, where the must is fermented by adding up to 100% of pomace named “*chacha*” (skins, pips, and stalks); ii) the “Imeretian style”, where the must is fermented in *qvevri* with partial (2.5–3.0%) addition of *chacha*; (iii) the so called “European style” without addition of *chacha*; (iv) the “Naturally semi-sweet wines” as well as sparkling wines that were also made in *qvevri* in the past (Ghlonti, 2010; NWA – National Wine Agency of Georgia, 2016). Because of the widespread of the “*qvevri* winemaking tradition” in Georgia, as proof of its cultural significance and in accordance with principles of Convention on Protection promoted by UNESCO, the status of National Monument of Intangible Cultural Heritage has been assigned to “The ancient Georgian tradition of *qvevri* winemaking” in 2013 (NWA – National Wine Agency of Georgia, 2016).

In recent years changes in the wine market have led to minor consumption in European countries, but with a strong demand toward health requirements and sensorial satisfaction. Currently the majority of wine production around the world is based on the use of starter cultures consisting of selected strains of yeasts (active dried yeast, ADY) and bacteria, that ensure quick and safe must transformation, reducing the risk of slow or stuck fermentation or spoilage due to microbial contamination. The practice of ADY inoculation, along with other technological innovations, has helped to improve wine quality by increasing the capability of winemakers to control the fermentation process and sensory profile. However, the low number of really different commercial strains often referred with different names (Fernandez-Espinar et al., 2001; Vigentini et al., 2015), has likely led a standardization of the product resulting in a taste leveling. This phenomenon requires the isolation and selection of new yeasts and bacteria showing technological, quality and safety features useful to obtain innovative products.

The work has aimed to explore the microbial biodiversity of a pristine environment that still represents a fascinating source for the isolation of new potential interesting strains, since it is a vine-growing area that has rarely been investigated before (Capece et al., 2013). Throughout the characterization and selection of indigenous yeasts isolated from oenological environments, our study has been addressed to improve quality of Georgian wines made from low aromatic local cultivars by exploiting the volatile compounds developed during fermentations. To obtain this goal, the dominant yeast populations present in 78 samples of grape and wine, from vineyards and traditional cellars located in five regions of Georgia, were analyzed during the 2014 vintage. In a perspective of precision oenology, three strains were chosen, on the basis on their oenological traits to perform vinifications experiments for the valorization of two widespread autochthonous grape varieties.

## Materials and Methods

### Yeast Sampling

Grape samples from 22 different native varieties were collected in 26 vineyards located in five regions, while wine samples were derived from 19 cellars of four regions (**Table 1**). Approximately 100 g of ripe bunches or 50 mL of wine at different stage of aging were taken, maintained at 4°C and transported in sterile bags to the laboratory. After crushing and homogenization by peristaltic apparatus (Stomacher 400, Colworth, UK) the obtained juice from the grape or the wine samples were decimally diluted in Peptoned Water (Merck, Germany); then, 100 μL of the appropriate dilutions were spread onto WL plates (Merck, Germany) that were incubated at 25°C for 3 days. Different type of colonies collected from the plates at the highest dilutions were streaked and purified twice on WL agar. The purified isolates were stored at –80°C in YPD broth (10 g/L yeast extract, 20 g/L peptone, 20 g/L glucose, pH 5.6) added with 20% (v/v) glycerol.

**Table 1:** Identification and distribution of yeast isolates in grape and wine samples for Georgian grape cultivars and geographic areas.

				*Aur. pullulans*	*Cand. gotoi*	*Cand. intermedia*	*Cry. carnescens*	*Cry. flavescens*	*Hans. guilliermondii*	*Hans. vineae*	*Kluy. marxianus*	*Met.a pulcherrima*	*Mey. guilliermondii*	*Pic. kluyveri*	*Pic. terricola*	*Sac. cerevisiae*	*Sta. bacillaris*	*Tor. delbrueckii*

**Grape cultivar**	**Area**	**Source**	**Vineyard/cellar**															
Aladasturi (b)	Guria	Grape	29,30				1	1	1			1						
Alexandreuli (b)	Ratcha-L.	Grape	20,36				1		1			2						
Asuretuli shavi (b)	Kartli	Grape	1,6						3						1			
	Kartli	Wine	24,25													4		
Chinuri (w)	Kartli	Grape	12,13					1	3		1	1						
		Wine	1,3,11,12,13, 26,27						2							11		
Chkapa (w)	Kartli	Grape	14	1								1						
		Wine	14													1	1	
Chkhaveri (r)	Guria	Grape	31				1	1								1		
Dzvelshavi (b)	Ratcha-L.	Grape	21						2			1		1				
Gorula (w)	Kartli	Grape	15					2				2						
Goruli Mtsvane (w)	Kartli	Grape	16	2	1													
Jani (b)	Guria	Grape	33					1				1				1		
Krakhuna (w)	Imereti	Grape	22					2	1					1				
		Wine	5						1							1	1	
Mtsvane Kakhuri (w)	Kakheti	Grape	2,3									4						1
		Wine	8													2		
Mtsvane Rachuli (w)	Ratcha-L.	Grape	23						2			2						
Mujuretuli (b)	Ratcha-L.	Grape	24					1			1	1						
Orbeluri Ojal. (b)	Ratcha-L.	Grape	25					1	1			1		1				
Otskhanuri Sap. (b)	Imereti	Grape	26,35			1			2			3						
Rkatsiteli (w)	Kakheti	Grape	5,7,8						3			3						2
		Wine	6,10,15,16,17,18,
			20,21,28,29,30,32						1			1	1			21		
	Kartli	Grape	4						2			1						
Saperavi (b)	Kakheti	Grape	9,10,11,17			1			2			6	1					2
		Wine	7,19,22,23,31,33,34						1							10		
Tavkveri (b)	Kartli	Grape	1,18,19	1		2		1	2			2						
		Wine	2,4						1							2		
Tsitka (w)	Imereti	Grape	27,34						3	1		2						
Tsolikouri (w)	Guria	Grape	37,38,39				2	1					1			1		
		Wine	9,35,36,37,38,39						1							9		
	Imereti	Grape	32				1									1		
Tsulukidzis (w)	Ratcha-L.	Grape	28						2			1						


*Berry color of the vine cultivar: b = black, r = rosé, w = white.*

### Yeast Identification

Yeast DNA was extracted according to Querol et al. (1992) protocol. The presumptive identification was attained by PCR amplification of the internal transcribed spacers between the 18S and 26S rDNA genes (ITS1-5.8S-ITS2) and subsequent restriction analysis according to Esteve-Zarzoso et al. (1999). The PCR mixture contained 1X *Taq* polymerase buffer with 1.5 mM MgCl_2_ (5 Prime, Hamburg, Germany), 1 mM MgCl_2_, 200 μM dNTPs (Fermentas, Vilnius, Lithuania), 0.1 μM of each primer ITSY1, and ITSY4 (Kurtzman and Robnett, 1998), 2 U *Taq*-DNA Polymerase (5 Prime) and 80–100 ng of DNA. The reaction was carried out in a T Gradient Biometra Thermocycler (Biometra, Göttingen, Germany) and the amplification was performed as follows: initial denaturation at 94°C for 5 min, then 35 cycles at 94°C for 1 min, annealing at 55°C for 1 min, and extension step at 72°C for 1 min, followed by final extension at 72°C for 7 min. PCR products were resolved by electrophoresis in 1.0% (w/v) agarose gels in TAE buffer (40 mM Tris–acetate, pH 8.2; 1 mM EDTA) at 100 V for 1 h, stained with 0.5 μg/mL ethidium bromide and photographed under UV illumination (GelDoc XR, BioRad, USA). A 100-bp XL DNA ladder marker (Roche Molecular Biochemicals, Mannheim, Germany) served as the size standard. Then, the amplified products were subjected to endonuclease restriction using 3U of *Hin*6I (Fermentas) according to the supplier’s instructions. Restriction fragments were resolved by electrophoresis in 2.5% (w/v) agarose gels in TAE buffer at 100 V for 2 h and detected as described above. Isolates showing the same restriction pattern were grouped and one or two samples per cluster was submitted to the partial amplification and sequencing of the 26S rDNA D1/D2 domain. The PCR mixture was prepared as mentioned above but with primer pairs NL1 and NL4 (Kurtzman and Robnett, 1998). The temperature profile consisted of initial denaturation at 94°C for 5 min then 35 cycles at 94°C for 1 min, annealing at 52°C for 1 min and extension step at 72°C for 2 min, followed by final extension at 72°C for 7 min. Amplification products were resolved by agarose gel electrophoresis as already described and then they were subjected to sequencing by an outdoor provider (Eurofins, Milan, Italy). The obtained sequences were identified through BLAST algorithm by comparison with the sequences listed in databases (www.ncbi.nlm.nih.gov).

### Evaluation of Oenological Traits

In order to select strains with oenological potential some phenotypic properties were investigated. Ethanol production was evaluated monitoring the weight loss for 2 weeks at 25°C in YPD modified adding 250 g/L glucose. As for the inoculum, 200 mL flasks containing 100 mL of cultural broth and sealed with a Müller trap, were inoculated at 0.25 OD_600 nm_. Acetic acid and glycerol production was determined by using specific enzymatic kits based on spectrophotometric UV-method according to the supplier’s recommendations (Megazyme International, Bray, Ireland). The resistance against sulfur dioxide was verified by observing the cellular growth of a fresh culture streaked onto YPD, supplied with 15 g/L agar and acidified at pH 3.6 with tartaric acid, after incubation at 25°C for 5 days. A stock sterile solution of potassium metabisulfite was added in the medium at the final concentration of 100, 200, and 300 mg/L. The production of hydrogen sulfide was phenotypically estimated streaking on BIGGY agar plates (Oxoid limited, Basingstoke, UK) a fresh culture and observing the color of the colonies after incubation at 25°C for 4 days.

### Vinification Experiments

On the basis of phenotypic results, *K. marxianus* (UMY207), *S. cerevisiae* (UMY255), *T. delbrueckii* (UMY196) were chosen for fermentation tests. Healthy and ripe grapes of Georgian local varieties Goruli Mtsvane (white cultivar) and Saperavi (black cultivar) were picked and manually selected to prepare the musts by moderate crushing of the berries. In case of Goruli Mtsvane must, the juice was clarified by cold settling at +4°C for 16 h, while for Saperavi must the juice was fermented together with skins and seeds. Diammonium phosphate (150 mg/L) and potassium metabisulphite (100 mg/L) were added. Vinification trials were carried out in a Georgian experimental cellar where the room temperature was recorded and set at approximately 20°C. As fermentation containers, 20 L high density polyethylene plastic carboys with stopper and airlock system were used. The inoculum was prepared in YPD broth at 25°C for 2 days with shaking. Fresh cells were collected by centrifugation at 4000 × *g* for 20 min, washed in sterile water and re-suspended in YPD. An aliquot of the cell suspension was added to the must in order to reach an initial concentration of 5 × 10^6^ CFU/mL. Fermentations were daily monitored by measurement of the sugar content with a refractometer (PR32 α digital refractometer, 3405 Palette Series, Atago, Tokyo, Japan) expressed as Brix degree. Samples were weekly collected for microbial and chemical analyses. pH value was measured by a pHmeter (UB-5 model, Denver Instruments Company, Bohemia, NY, USA); titratable acidity (expressed as g/L of tartaric acid) was determined by titration of the juice with 0.1 N NaOH with Bromothymol blue as the indicator. Yeast count, isolation and identification of some colonies at the highest dilutions were done as described in the previous paragraphs in order to control the trend of inoculated yeasts.

### Chemical Analysis of Volatile Compounds

Volatile compounds present in must and wine samples during the vinification tests were quantified using the method proposed by Ortega et al. (2001), modified as follows. 2.5 grams of ammonium sulfate and 2 mL of wine were mixed in a 15 mL centrifuge tube. Five mL of ultrapure water and 20 μL of internal standards were added to the solution. The standard mixture consisted of a working solution (50% ethanol) containing 2-butanol (0.948 mg/L), 4-methyl-2-pentanol (0.940 mg/L), ethyl heptanoate (1.077 mg/L), heptanoic acid (1.102 mg/L), 4-hydroxy-4-methyl-2-pentanone (1.145 mg/L), 2-octanol (0.945 mg/L). After salt dissolution, 250 μL of dichloromethane were added to the samples. Tubes were placed on a horizontal stirrer with a speed of approximately 60 rpm/min for 90 min; at the end of the extraction, samples were centrifuged (4000 × *g*, 10 min, 10°C). The supernatants were discarded, while the dichloromethane containing the analytes was removed with a 250 μL syringe, dehydrated with sodium sulfate and placed in 2 mL vials with 250 μL inserts. The analyses were performed with a Hewlett Packard 5890 II series GC-FID in splitless mode using a polar capillary column (HP Innovax, 30m × 0.25mm ID 0,25 μm JW Scientific, Folsom, CA, USA); the carrier gas was helium with a column flow of 1 mL/min and a splitless time of 2 min. The program was as follows: 45°C for 2 min, then the temperature was increased to 80°C at 30°C/min, from 80°C to 230°C at 5°C/min and held at 230°C for 17 min. All compounds were identified by comparison with the retention time of pure standards injected in the same chromatographic conditions.

Samples were analyzed after 6 months of storage. Twenty-four aromatic molecules were quantified, belonging to the following five groups: higher alcohols, ethyl esters, short and medium chain fatty acids, ester acetates of higher alcohols and a miscellaneous group, comprising other volatiles such as (3-hydroxybutan-2-one or acetoine, (3Z)-hex-3-en-1-ol, 1-hexanol, dihydrofuran-2(3H)-one, or γ-butyrolactone and phenylmethanol or benzyl alcohol. Duplicate analyses were performed for all samples.

### Data Analysis

The frequency (*F*’) and the incidence (*I*’) of the yeast species in the grape samples were calculated according to Tristezza et al. (2013). Significant differences among wine samples analyzed at the same sampling point during fermentations were assessed by one-way ANOVA, for each groups of odorants described before. Differences among means were evidenced by using the Tukey’s test and were considered significant at *p* < 0.05. Differences were represented by different letters on the graph. In order to compare yeast strain and grape must effect treatments on the final wines, two-way ANOVA was performed for each analyzed volatile compounds. Statistical analyses were performed with the software package SPSS (SPSS 15.0 for Windows 2004; SPSS, Chicago, IL, USA).

## Results

### Evaluation of Yeast Biodiversity

The mean value of the yeast concentration on grape samples was 2.946 log CFU/g (±1.240, SD), whereas that found in wine samples was 6.026 log CFU/mL (±1.712). One hundred and eighty two isolates, 110 from 39 grape samples recovered from 22 Georgian autochthonous *Vitis vinifera* cultivars and 72 from 39 wine samples collected from 19 cellars were ascribed to 15 different taxa by RFLP analysis of ITS region and the partial sequencing of 26S rDNA gene (**Table 1**). In particular, the yeasts most frequently isolated on grapes and with the highest incidence were *Metschnikowia pulcherrima* (*F*’ = 0.56, *I*’ = 0.32), *Hanseniaspora guilliermondii* (*F*’ = 0.49, *I*’ = 0.27), *Cryptococcus flavescens* (*F*’ = 0.31, *I*’ = 0.11), and *Cryptococcus carnescens* (*F*’ = 0.13, *I*’ = 0.05). Other taxa found with a lower frequency and incidence were *Torulaspora delbrueckii* (*F*’ = 0.10 *I*’ = 0.05), *Aureobasidium pullulans* (*F*’ = 0.10, *I*’ = 0.04), *Candida intermedia* (*F*’ = 0.08, *I*’ = 0.04), *Saccharomyces cerevisiae* (*F*’ = 0.08, *I*’ = 0.04)*, Pichia kluyveri* (*F*’ = 0.08, *I*’ = 0.03), *Meyerozyma guilliermondii* (*F*’ = 0.05, *I*’ = 0.02), *Kluyveromyces marxianus* (*F*’ = 0.05, *I*’ = 0.02), *Candida gotoi* (*F*’ = 0.03, *I*’ = 0.01), *Hanseniaspora vineae* (*F*’ = 0.03, *I*’ = 0.01), and *Pichia terricola* (*F*’ = 0.05, *I*’ = 0.01). As expected, *Saccharomyces cerevisiae* was dominant in wine samples since its presence was determined in 97% of cases, confirming the key role of this species in Georgian spontaneous fermentations. However, given that the samples were collected at different stage of aging, a certain biodiversity has been observed including few other species, such as *H. guilliermondii* which was present in seven samples, *Starmerella bacillaris* in two samples, *M. pulcherrima* and *M. guilliermondii* in one sample. These results are generally in accordance with those recently reported by some authors in similar survey activities (Zott et al., 2010; Barata et al., 2012; Vigentini et al., 2015).

### Strain Selection by Oenological Traits

**FIGURE 1:**
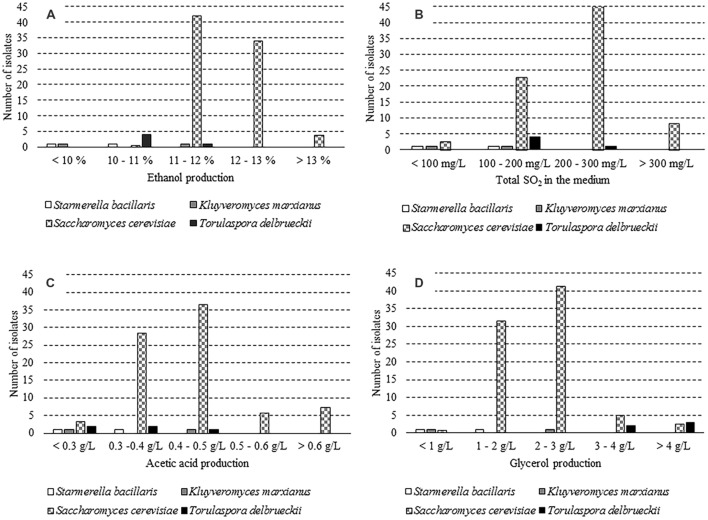
**Distribution of the isolates ascribed to four yeast species for some oenological traits.** **(A)** Differences in ethanol production, **(B)** Differences in sulphur dioxide resistance, **(C)** Differences in acetic acid production, and **(D)** Differences in glycerol production.

Seventy-four out of 182 isolates were sorted on the supposed ability of a fermentative metabolism. In particular, 65 clones of *S. cerevisiae*, 5 of *T. delbrueckii*, 2 of *S. bacillaris*, and 2 of *K. marxianus* were investigated for some oenological traits. As expected, *S. cerevisiae* has proved to be the species with the highest ethanol production (**Figure 1A**). Four *T. delbrueckii* isolates grew up to 10–11% v/v, while one strain reached an alcohol content of 11.5% v/v. Only one isolate of both *K. marxianus* and *S. bacillaris* species was able to produce alcohol between 10 and 11% v/v. Growth tests for sulfur dioxide resistance revealed that most *S. cerevisiae* isolates (69%) withstood up to 200 mg/L of total SO_2_, whereas *K. marxianus* and *S. bacillaris* isolates did not (**Figure 1B**). The determination of acetic acid concentration has evinced that *S. cerevisiae* exhibited a heterogeneous behavior, with 17% isolates that produced high amount of this compound (>0.5 g/L). The two S. *bacillaris* strains displayed an acetic acid production lower than 0.4 g/L, while *K. marxianus* and *T. delbrueckii* isolates revealed dissimilar capabilities (**Figure 1C**). Additionally, it was found that 91% of *S. cerevisiae* isolates yielded lesser than 3 g/L of glycerol. *T. delbrueckii* showed a good performance since three isolates out of five (60%) produced a glycerol concentration higher than 4 g/L. On the other hand, *K. marxianus* and *S. bacillaris* strains exposed a lesser capacity to synthesize this compound (**Figure 1D**). Results of the qualitative test on the hydrogen sulfide development showed that 53 *S. cerevisiae* isolates (82%) were high producers (brown colonies), 9 isolates (14%) were low producers (light brown colonies) and only 3 isolates (5%) did not produce it (white colonies). *T. delbrueckii* isolates proved to be low producers, as well as one strain of *K. marxianus* and of *S. bacillaris*; the remaining ones for both species were high producers of hydrogen sulfide. These findings are mostly in agreement with those reported by some authors for non-*Saccharomyces* yeasts (Lambrechts and Pretorius, 2000; Cordero-Bueso et al., 2013; Englezos et al., 2015). The comparison of results obtained from the previous tests allowed to select three yeast strains, *K. marxianus* UMY207, *S. cerevisiae* UMY255, and *T. delbrueckii* UMY196 with oenological potential for the vinification experiments.

### Monitoring of the Vinification Experiments

**FIGURE 2:**
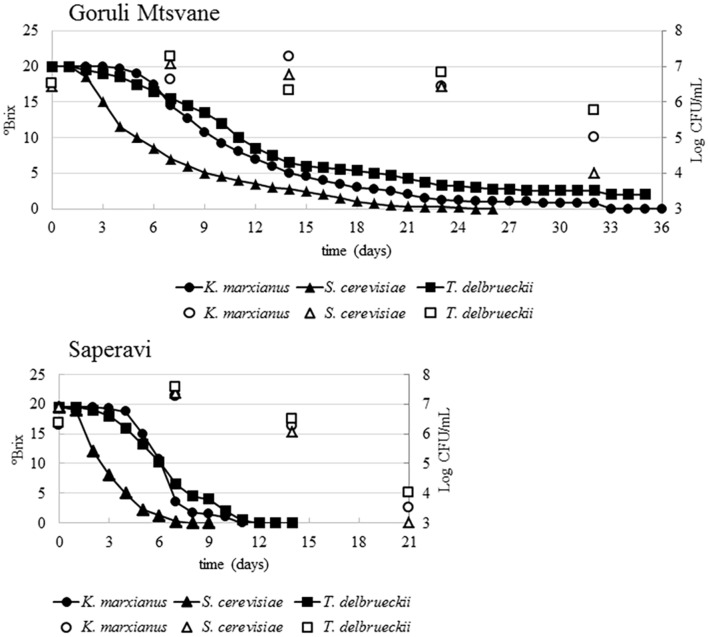
**Trends of sugar concentrations (lines and filled dots) and viable counts (unfilled dots) during the fermentation of Goruli Mtsvane (Georgian white berry cultivar) and Saperavi (Georgian black berry cultivar) musts inoculated with *Kluyveromyces marxianus* UMY207, *Saccharomyces cerevisiae* UMY255, and *Torulaspora delbrueckii* UMY196 strains**.

Fermentations trials were carried out for each selected strain that were separately inoculated in 20 L volume musts of two Georgian grape cultivars (Goruli Mtsvane and Saperavi). All operations were rigorously done in strictly hygienic conditions to avoid cross-contamination between the strains. The temperature of the cellar was comprised between 17.5 and 21.0°C, with a mean value of 19°C. Chemical analysis and microbial counts were weekly scheduled and performed to monitor the fermentative process. The initial pH value and the titratable acidity of Goruli Mtsvane must were 3.1 and 7.6 g/L, respectively; the initial pH value and the titratable acidity of Saperavi must were 3.2 and 6.9 g/L. The yeast count of the non-inoculated white grape must was 1.1 × 10^4^ CFU/mL, whereas that of the non-inoculated black grape must was 1.3 × 10^5^ CFU/mL. For all the tested strains, in Saperavi must the fermentation started and ended earlier than in Goruli Mtsvane must (**Figure 2**). The trend of sugars consumption (Brix) revealed that the lag phase in Saperavi must inoculated with *S. cerevisiae* UMY255 was 24 h, while those with *T. delbrueckii* UMY196 and *K. marxianus* UMY207 lasted until 2 and 3 days, respectively. Then, the sugar depletion went on with different rates depending on the tested strain, so that one inoculated with *S. cerevisiae* UMY255 completed the fermentation within 8 days, while the trial inoculated with *T. delbrueckii* UMY196 took 10 days and finally that one inoculated with *K. marxianus* UMY207 needed 14 days. After a week, yeast counts varied from 3.7 × 10^7^ CFU/mL, in Saperavi must inoculated with *T. delbrueckii* UMY196, to 1.8 × 10^7^ CFU/mL, in that inoculated with *K. marxianus* UMY207. At that time, the identification of the dominant populations revealed that 100% *S. cerevisiae*, 100% *T. delbrueckii*, and 60% *K. marxianus* were present, as expected, into the relative vessels. After two weeks the yeast cell concentrations decreased, ranging from 3.1 × 10^6^ CFU/mL in the trial inoculated with *T. delbrueckii* UMY196, to 1.1 × 10^6^ CFU/mL in that inoculated with *S. cerevisiae* UMY255. At this point, in the vessels inoculated with *T. delbrueckii* and *K. marxianus* only 25 and 10%, respectively, of the colonies isolated at the highest dilutions corresponded to the predictable species; conversely, for the trial inoculated with *S. cerevisiae*, 100% was ascribed to the expected species. Then, yeast counts dropped <10^4^ CFU/mL after twenty days. The fermentation kinetics in Goruli Mtsvane must appeared otherwise, although the cell concentrations after the inocula were very similar to those obtained in Saperavi must and constantly >10^6^ CFU/mL. The lag phase of the sample inoculated with *S. cerevisiae* UMY255 lasted 2 days, whereas for those with *T. delbrueckii* UMY196 and *K. marxianus* UMY207 it persisted until 3 and 4 days, respectively. Then, sugars consumption rates were much lower than those previously observed: the fermentation of Goruli Mtsvane must inoculated with *S. cerevisiae* UMY255 finished in three weeks, while the trials inoculated with the other strains did not complete the transformation even after a month, leaving some residual sugars (1–3 °Brix). In particular, after a week, yeast cell concentrations varied from 1.9 × 10^7^ CFU/mL (in must inoculated with *S. cerevisiae* UMY255) to 4.2 × 10^6^ CFU/mL (in that one with *K. marxianus* UMY207). The dominant populations were attributed to 100% *S. cerevisiae*, 86% *T. delbrueckii* and 100% *K. marxianus*, matching to the inoculated species. After 2 weeks, yeast counts passed from 1.8 × 10^7^ CFU/mL (in must inoculated with *K. marxianus* UMY207) to 2.1 × 10^6^ CFU/mL (in that one with *T. delbrueckii* UMY196); but, from this point on, *K. marxianus* was not found, while *T. delbrueckii* and *S. cerevisiae* represented 50 and 100% of the fermentative biomass in the relative vessels, respectively. Yeast counts approximately decreased to 10^5^ CFU/mL after one month. The identification of the isolates at the end of the fermentation showed the dominance of other yeasts, mainly ascribed to *S. cerevisiae* species, which naturally contaminated the musts.

Samples weekly collected were also subjected to analysis GC-FID analysis in order to quantify the most relevant volatile compounds. Some of them, in particular aromas resulting from yeast fermentation (i.e., ethyl esters of short and medium fatty acids) are responsible for the pleasant fresh fruity notes perceived in wines.

**FIGURE 3:**
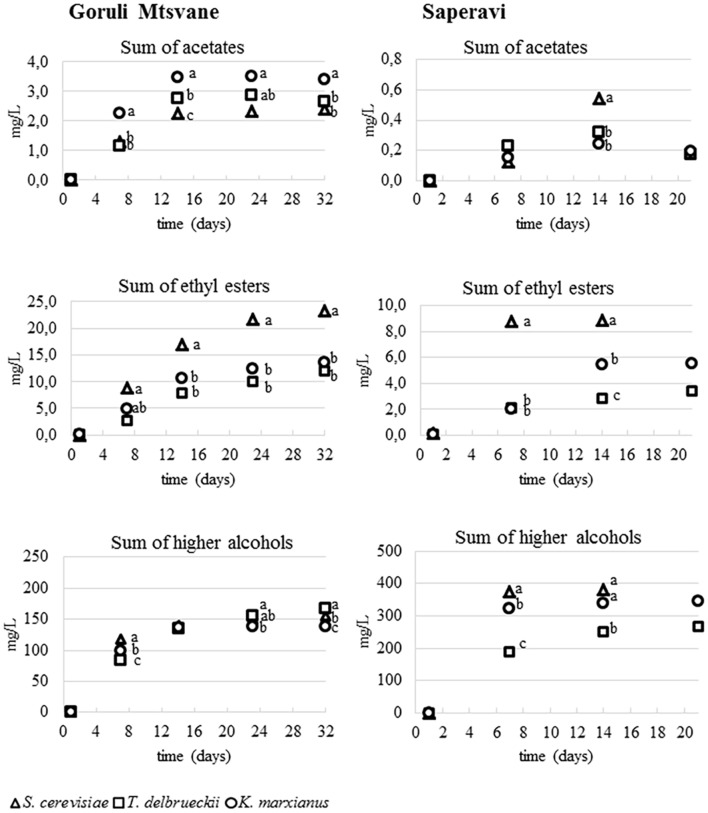
**Monitoring of the concentrations of three classes of volatile compounds during the fermentation of Goruli Mtsvane (Georgian white berry cultivar) and Saperavi (Georgian black berry cultivar) musts inoculated with *K. marxianus* UMY207, *S. cerevisiae* UMY255 and *T. delbrueckii* UMY196 strains.** Dots with different letters at the same time indicate mean values significantly different (*p* < 0.01).

**Table 2** reports the mean concentrations of the volatile compounds found in wines inoculated with different strains (*S. cerevisiae* UMY255, *T. delbrueckii* UMY196, *K. marxianus* UMY207) or in wines obtained from diverse grape varieties (Saperavi and Goruli Mtsvane). The concentrations of the main higher alcohols, 2-methylpropan-1-ol, 3-methylbutan-1-ol, and 2-phenylethanol, were comparable with those reported by Swiegers et al. (2005), since the sum of these ranged from 130 to 300 mg/L. In particular, *S. cerevisiae* UMY255 was the strain by revealing the greatest yield (241 mg/L). On the contrary the mean concentration of the higher alcohols in wines fermented by *T. delbrueckii* UMY196 (191.5 mg/L) was significantly (*p* < 0.001) lower than the others, showing small contents of 2-methylpropan-1-ol and 2-phenylethanol. These results are in agreement with Renault et al. (2009), who observed a low quantity of higher alcohols in wines fermented with *T. delbrueckii.* However, the composition of the musts can significantly influence the synthesis of aromatic compounds by different yeasts, as significant interactions were pointed out (**Table 2**). High levels of 2-methylpropan-1-ol, 3-methylbutyl acetate, and 2-phenylethanol were produced by *K. marxianus* UMY207 and *S. cerevisiae* UMY255 in Saperavi wine samples (**Figure 3**). This trend was not noticed in that fermented with *T. delbrueckii*. On the other hand, the concentration of these compounds in Goruli Mtsvane wines were higher for those inoculated with *T. delbrueckii* rather than the samples obtained using the other yeast strains. Some remarkable differences regarding the wine composition are related to the ethyl esters production. Actually, the production of ethylhexanoate, ethyloctanoate, and ethyldecanoate proved to be significantly greater in wines inoculated with S. *cerevisiae* UMY255 than in those inoculated with *T. delbrueckii* UMY196 and *K. marxianus* UMY207 ones (**Table 2**). These findings confirm the results reported by Renault et al. (2009) where cultures of *T. delbrueckii* have been shown to produce low levels of ethyl esters. Nevertheless, high concentrations of both 4-ethoxy-4-oxobutanoic acid and ethyl 2-hydroxypropanoate were detected in wine samples performed by inoculating *T. delbrueckii* UMY196 and *K. marxianus* UMY207 (about two folds higher than in musts inoculated with *S. cerevisiae* UMY255). 4-ethoxy-4-oxobutanoic acid concentration in white wines appeared significantly lower than that found in red wines (78.7 mg/L vs. 17.4 mg/L, *p* < 0.001). As concerns the corresponding diethylester, the diethyl butanedioate, is normally generated during wine storage, therefore its content is usually low in young wines and the differences between experimental tests limited. Interactions between yeast and must may affect the formation of these esters, namely the 4-ethoxy-4-oxobutanoic acid and ethyl 2-hydroxypropanoate. In Saperavi musts, inoculated with *T. delbrueckii* UMY196 and *K. marxianus* UMY207, a greater accumulation of ethyl esters was found (51.5 and 49.2 mg/L, respectively) than that observed in trials inoculated with *S. cerevisiae* (14.0 mg/L). As regards the short and medium chain fatty acids, the concentrations of hexanoic and octanoic acids were significantly higher (*p* < 0.001) in white wines rather than red wines, while no statistical differences were detected for decanoic acid. Yeast related differences were modest and concerned only the 1-hexanoic acid (*p* < 0.05, **Table 2**). On average, the concentration of these compounds was higher in the wines obtained from *S. cerevisiae* fermentations. Finally, it is noteworthy the higher concentration of acid 3-methylbutanoic acid in Saperavi in wines than the corresponding Goruli Mtsvane wines (**Table 2**). The concentrations of the ester acetates of higher alcohols determined in samples fermented with different yeasts, appeared similar. A greater production of these compounds was noticed in musts inoculated with *S. cerevisiae* UMY255, and significant differences (*p* < 0.001) were shown between white and red wines for the 3-methylbutyl acetate (0.88 mg/L in Goruli Mstvane vs. 0.17 mg/L in Saperavi) and the phenylethylacetate contents. Concerning the others compounds, dihydrofuran-2(3H)-one presented high concentrations in wines fermented with *S. cerevisiae*. 1-Hexanol and (3Z)-hex-3-en-1-ol showed minor yeast related differences, while statistically significant changes were pointed out for 1-hexanol between Saperavi and Goruli Mtsvane wines (**Table 2**). This outcome confirms that the presence in wines of alcohols with six carbon atoms are mainly due both to the grape variety and oxygen uptake during pre-fermentative operations (Oliveira et al., 2006).

**Table 2:** Volatile composition of experimental wines and perception threshold of main odorants.

Volatile compound	Odorant (1, 2)	Perception threshold	Yeast strain (Y)			Grape cultivar (Cv)				Interaction
					
			*S. cerevisiae* UMY-255	*T. delbrueckii UMY-196*	*K. marxianus* UMY-207		Goruli mtsvane	Saperavi		Y x Cv
**Higher alcohols**										
2-Methylpropan-1-ol (isobutanol)	Ethereal	40^(3)^	38^a^	27^b^	38^a^	^∗∗∗^	21.6	47	^∗∗∗^	^∗∗∗^
3-Methylbutan-1-ol (isoamyl alcohol)	Fusel oil	30^(3)^	182^a^	149^b^	152^b^	^∗∗∗^	100	222	^∗∗∗^	^∗∗∗^
2-Phenylethanol	Rose	14^(4)^	21.3^b^	15.5^c^	25.0^a^	^∗∗∗^	9.4	31.7	^∗∗∗^	^∗∗∗^
3-(Methylsulfanyl) propan-1-ol (methionol)	Onion-like	1^(3)^	2.0^a^	1.3^b^	1.4^b^	^∗∗^	0.3	2.8	^∗∗∗^	^∗∗^
**Ethyl esters**										
Ethyl hexanoate	Strawberry	0.014^(4)^	1.3^a^	0.7^b^	0.9^b^	^∗∗∗^	1.1	0.8	^∗∗^	^∗∗^
Ethyl 2-hydroxypropanoate (ethyl lactate)	Fruity	154^(5)^	11.8^c^	27.4^b^	29.2^a^	^∗∗∗^	7.4	38.2	^∗∗∗^	^∗∗∗^
Ethyl octanoate	Soap	0.005^(4)^	0.5^a^	0.3^b^	0.4^ab^	^∗^	0.5	0.2	^∗∗∗^	n.s.
Ethyl 3-hydroxybutanoate (ethyl-3-hydroxybutyrate)	Fruity	20^(3)^	0.9^a^	0.3^b^	0.3^b^	^∗∗∗^	0.3	0.7	^∗∗∗^	^∗∗∗^
Ethyl 4-hydroxybutanoate (ethyl-4-hydroxybutyrate)	Apple	-	2.3^a^	1.5^ab^	1.1^b^	^∗^	1.6	1.6	n.s.	n.s.
Ethyl decanoate	Grape	0.2^(4)^	0.3^a^	0.3^a^	0.1^a^	n.s.	0.3	0.2	n.s.	n.s.
Diethyl butanedioate (diethyl succinate)	Fruity	200^(5)^	0.9^a^	0.6^b^	0.8^a^	^∗∗∗^	0.8	0.8	n.s.	^∗∗^
4-Ethoxy-4-oxobutanoic acid (monoethyl succinate)	Cooked apple	-	27^b^	58^a^	59^a^	^∗∗∗^	17.4	78.7	^∗∗∗^	^∗∗∗^
**Short and medium chain fatty acids**								
Hexanoic acid	cheesy	0.42^(4)^	1.8^a^	1.5^b^	1.5^b^	^∗^	2.2	1.0	^∗∗∗^	^∗∗∗^
Octanoic acid	rancid	0.5^(4)^	1.7^a^	1.3^a^	1.5^a^	n.s.	2.4	0.5	^∗∗∗^	^∗^
Decanoic acid	rancid	1^(4)^	0.4^a^	0.3^a^	0.3^a^	n.s.	0.3	0.3	n.s.	n.s.
3-Methylbutanoic acid (isovaleric acid)	cheesy	0.03^(4)^	1.2^b^	3.5^a^	3.8^a^	^∗∗∗^	0.3	5.3	^∗∗∗^	^∗∗∗^
**Ester acetates of higher alcohols**									
Hexyl acetate	green apple	1.5^(5)^	0.2^a^	0.1^b^	0.1^b^	^∗∗^	0.1	0.1	n.s.	^∗^
3-Methylbutyl acetate (isoamyl acetate)	banana	0.03^(4)^	0.8^a^	0.2^b^	0.5^b^	^∗∗∗^	0.9	0.2	^∗∗∗^	^∗∗^
2-Phenylethyl acetate	rose, honey	0.25^(4)^	0.01^b^	0.01^b^	0.06^a^	^∗∗∗^	0.05	0.0	^∗∗∗^	^∗∗∗^
**Miscellaneous group**										
3-Hydroxybutan-2-one (acetoine)	buttery	150^(5)^	0.6^c^	1.2^b^	1.8^a^	^∗∗∗^	1.1	1.3	^∗∗∗^	^∗∗∗^
(3Z)-Hex-3-en-1-ol (cis-3-Hexenol)	grassy	0.1^(4)^	0.04^a^	0.03^b^	0.03^b^	^∗∗∗^	0.03	0.03	n.s.	^∗^
Hexan-1-ol	herbal	8^(3)^	1.5^a^	1.2^a^	1.3^a^	n.s.	0.7	2.0	^∗∗∗^	n.s.
Dihydrofuran-2(3H)-one (γ-butyrolactone)	creamy	100	7.2^a^	4.1^b^	3.8^b^	^∗∗∗^	5.6	3.9	^∗∗∗^	^∗∗^
Phenylmethanol (benzyl alcohol)	floral	200^(3)^	0.12^b^	0.14^a^	0.13^ab^	^∗^	0.05	0.2	^∗∗∗^	^∗∗∗^


*All the values are expressed in mg/L; n.s. = not significant; ^∗^ = ANOVA, *p* ≤ 0.05; ^∗∗^ = ANOVA, *p* ≤ 0.01; ^∗∗∗^ = ANOVA, *p* ≤ 0.001. (1) Cordero-Bueso et al. (2013); (2) Vilanova et al. (2013); (3) Guth (1997); (4) Ferreira et al. (2000); (5) Etiévant (1991). Values are grouped by yeast strain (Y) and grape cultivar (Cv). Values on the same line with different letter superscripts are significantly different.*

### Volatile Compounds Development during Fermentations

**Figure 3** shows the accumulation kinetics of three different classes of compounds during the trials (acetates of higher alcohols, ethyl esters, and higher alcohols), for both white and red musts inoculated with the different yeast strains. The average concentration of acetates in white samples was significantly higher than that found in the red ones. As reported in literature (Plata et al., 2003), the content of acetates of higher alcohols in wine depends substantially on the yeast strain and the interactions between yeast and grape must as well as the fermentation conditions (Molina et al., 2007). In Goruli Mtsvane samples the total concentrations of acetates increased until 2 weeks, then values remained constant; the highest content was found in that inoculated with *K. marxianus* UMY207. In general, the levels of ethyl esters increased steadily during all fermentations. According with some authors (Renault et al., 2009; Sumby et al., 2010) musts added with *S. cerevisiae* cultures produced the greatest amounts of these compounds respect those in which other yeasts were used. As regards the most odorant ethyl esters (ethyl hexanoate, ethyl octanoate, ethyl decanoate), the differences between the yeast strains during the fermentation were less evident (data not reported). However, *T. delbrueckii* UMY196 showed a small accumulation of these compounds when compared to *S. cerevisiae* and *K. marxianus*. These results are in agreement with those obtained recently by Hernandez-Orte et al. (2008) and Viana et al. (2008). Also in this case, the grape variety affected the ethyl esters production, since the white wine samples presented a double amounts of these fruity compounds compared to red ones. Higher alcohols increased rapidly during the fermentation; these molecules reached their maximum level after three weeks for the Goruli Mtsvane samples and at about the 14th day for the Saperavi ones, then a slight decrease was observed in both trials. The final concentration in wines strictly depended on the interaction between grape must and yeast strain. Indeed, at the end of the fermentation, the greatest amount of the higher alcohols was observed in red wine samples inoculated with *S. cerevisiae* UMY255 and *K. marxianus* UMY207.

## Discussion

While the precision viticulture is currently applied to optimize the vineyards performance in maximizing grape yield and quality, the precision oenology, that might exploit the technological potential of wild strains, still remains a matter of research activities. However, new styles of wine products and innovative ways of fermentation management have intensified the interest in search for new strains hidden in the microbial diversity (Pretorius and Bauer, 2002; Romano et al., 2003; Fleet, 2008; Jolly et al., 2014). Indeed, the best expression of the varietal character of a wine may depend on the metabolic activities of microorganisms taking part in the transformation of must and in the aging of wine (Lambrechts and Pretorius, 2000; Swiegers et al., 2005; Hernandez-Orte et al., 2008; Furdikova et al., 2014). Moreover, several research groups have recently addressed their efforts to collect and characterize “autochthonous” yeast strains as strategic activity for the promotion and protection of local wines, since these findings would confirm the link among territory, environment production and final product, with a remarkable commercial impact (Mannazzu et al., 2002; Di Maio et al., 2012; Settanni et al., 2012; Tristezza et al., 2012; Rodriguez-Palero et al., 2013). So, this study have given the opportunity of isolating novel wine-associated yeasts from Georgia, an ancient vine-growing area where the use of starter cultures has not yet spread, in order to select non-conventional yeast strains and species for wine-making.

The results obtained from grape samples have revealed a high level of biodiversity with rates of isolation and yeast species similar to those already described by different authors (Cordero-Bueso et al., 2011; Barata et al., 2012; Bokulich et al., 2013; Milanovic et al., 2013; Vigentini et al., 2015). No evident relationship has appeared between the yeast species and the grape cultivars or the geographic region of isolation, even if the number of isolates per sample was too small for drawing definitive conclusions. The high rate of isolation (10%) of *S. cerevisiae* species from the grape berries and the presence of *K. marxianus* have been interesting outcomes. The occurrence of the yeast species observed in Georgian wine samples are different from those reported by Capece et al. (2013), who only found *S. cerevisiae* species. However, these authors analyzed wines of a unique grape variety, from only one winery, after 1 year maturation in *qvevri* vessels, while we have sampled wines at different stages of aging, not only aged in clay amphorae, from 19 cellars and made with different grape cultivars. This may explains the isolation of other yeast species, such as *H. guilliermondii* and *S. bacillaris*; as well, the presence of other taxa besides *S. cerevisiae* has already been observed in wines from spontaneous fermentations (Torija et al., 2001; Combina et al., 2005; Zott et al., 2010; Vigentini et al., 2014). With regard to the screening activity for the strain selection with oenological potential *S. cerevisiae* has shown an intraspecific variability in phenotypic traits and, as expected, it has revealed the highest ethanol production and sulfur dioxide tolerance (Ribéreau-Gayon et al., 2006). *T. delbrueckii* strains have proved to be low producers of volatile acidity and good producers of glycerol, confirming the results reported by some authors (Bely et al., 2008; Renault et al., 2009); indeed, this species is currently the most applied in commercial starter cultures as non-*Saccharomyces* yeast for mixed fermentation or sequential inoculation technique (Jolly et al., 2014; Loira et al., 2014). *S. bacillaris* isolates (synonym of *Candida zemplinina*) have shown low rate of isolation and they have not demonstrated high performances in terms of glycerol production or alcohol resistance, respect to previous outcomes (Sadoudi et al., 2012; Englezos et al., 2015); for this reason, they were not taken into consideration for the vinification experiments. Conversely, although in a very limited number, *K. marxianus* strains have been considered appealing since they exhibited promising phenotypic traits for the application in wine-making. Due to its inherent ability to produce abundant quantities of esters, this species is emerging as a model organism to produce flavor compounds (Morrissey et al., 2015). However, in our experimental conditions, *K. marxianus* UMY207 has pointed out a scarce fermentation power and its presence has detected only in the first days of fermentation, and then overcome by the wild yeasts.

The choice of Goruli Mstvane and Saperavi varieties to be tested in the fermentation trials has been determined because they are two of the most cultivated in Georgia and they are low aromatic grapes cultivars, suitable to better show the ability of producing fermentative aromas by the selected strains (Viana et al., 2008; Romano et al., 2014). Indeed, higher alcohols, ethyl esters of short and medium fatty acids and acetates of higher alcohols, have been considered and monitored during the experimental fermentations, being responsible for the pleasant fresh fruity notes perceived in wines. The quantities of higher alcohols obtained in our trials are remarkable and encouraging since they show significant differences in the interactions between inoculated strain and grape variety. Instead, the findings about formation of ethyl esters and acetates of higher alcohols, have been less satisfactory, because most of these volatile compounds were quantified under their perception threshold.

A first reached goal of this study is the microbial collection that represents a contribution to the preservation and valuation of Georgian viti-oenological resources, found in a territory dedicated for thousands years to the wine production through traditional practices. As second target, our findings have highlighted that the production of volatile compounds significantly depends from the interaction between the grape cultivar and the yeast strain or species inoculated. Further vinification experiments in larger volumes with mixed cultures (by co-inoculation or sequential inoculation) should be performed to confirm the positive role of the selected Georgian yeast strains. In general, the outcomes of this work can be regarded as an advancement in the field of wine-making in order to edit the wine quality in a perspective of precision oenology for which the suitable grape cultivar is associated with the skillful yeast.

## Author Contributions

IV contributed to the design of the work, to the molecular identification of yeasts and the selection of candidates for vinification experiments, to the interpretation of data for the work, to draft the work and revising it; DM contributed to the design of the work, to collect grape and wine samples, to isolate yeasts and to manage vinifications; MP contributed to the chemical analysis of wine samples, to the interpretation of data for the work and to draft the work; FB contributed to the chemical analysis of wine samples, to the interpretation of data for the work and to draft the work; VM contributed to the preparation of musts for winemaking and to monitor chemical and microbiological analysis of vinifications; FV contributed to the yeast isolation and molecular identification of yeasts; OF contributed to the organization of the group and to draft the work; RF contributed to the design of the work, to the acquisition, the analysis of data for the work, to draft the work and revising it, and ensured that that questions related to the accuracy or integrity of any part of the work were appropriately investigated and resolved.

## Conflict of Interest Statement

The authors declare that the research was conducted in the absence of any commercial or financial relationships that could be construed as a potential conflict of interest.
